# Role for Isolated Emergency Medicine Physicians During a Pandemic

**DOI:** 10.1017/cem.2020.390

**Published:** 2020-05-05

**Authors:** Daniel Rosenfield, Jordan Levinter, Trent Mizzi, Greg Harvey

**Affiliations:** * Division of Paediatric Emergency Medicine, Department of Paediatrics, Hospital for Sick Children & University of Toronto, Toronto, ON

**Keywords:** Medical informatics, pediatrics, public health

## BACKGROUND

The coronavirus disease 2019 (COVID-19) pandemic has created unique staffing pressures on emergency departments (EDs) as a result of many staff being required to self-isolate due to travel, symptoms, contact, or proven COVID-19 infection. In addition to those staff who are unable to work due to self-isolation, our Division of Paediatric Emergency Medicine decided to insulate our at-risk staff physicians who were over 60 years of age, had significant comorbidities, or were pregnant. Understandably, during a health care crisis, the loss of these valuable staff members shifts the burden onto the remaining frontline health care providers. Additionally, the displacement and sudden lack of perceived purpose can be frustrating to those who are used to providing direct clinical care and who want to help with the crisis. While virtual care has been heralded as a solution in many outpatient environments, it is more challenging in an ED because the clinician has no prior rapport with the patient, the patient problems are typically higher risk, and the physical exam component of a patient assessment is often essential to making an accurate diagnosis.

**Figure 1.**
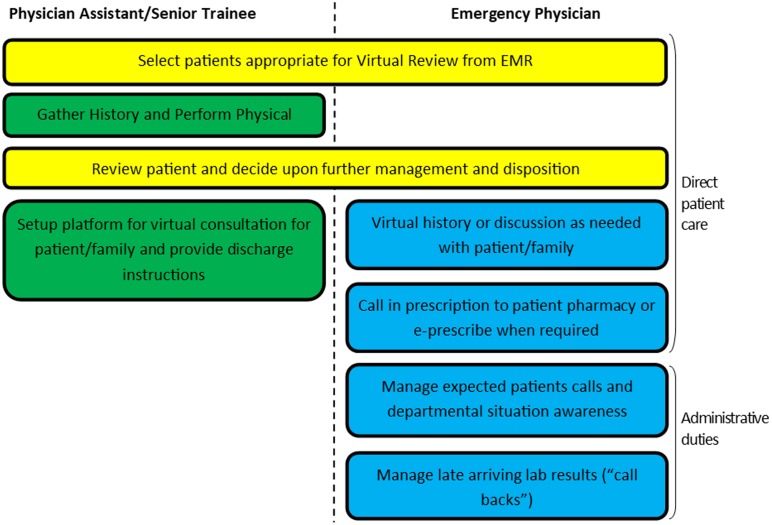
Roles of physician assistants and virtual attendings in the ED virtual review model.

This article describes the different roles that an isolated clinician can assume while self-isolating as a “virtual attending.” Our tertiary care children's ED went live with virtual reviewing on March 20, 2020, and has been making iterations to it since its launch. This was relatively straightforward to achieve from a funding perspective as our department has an alternate funding plan model. Unlike true virtual care where there is no expectation of a physical examination, the patients being reviewed virtually have undergone a triage assessment with documentation of vital signs, and a physical examination by a physician assistant (PA). In addition to helping with patient flow, our virtual attending has also helped to offload the administrative burden of the on-site team by taking outside phone calls, managing late-arriving test results, and handling discrepant radiology reports. By managing multiple issues from off-site, directly and indirectly valuable personal protective equipment (PPE) is also conserved. Undertaking these tasks from home is facilitated by a robust electronic medical record (EMR) system that can be accessed remotely. Roles in the virtual review model are summarized in Figure 1.

The following represents a description of how we have operationalized our virtual attending. While not every ED will necessarily be able to match the workflows described below, they can assess their own strengths and weaknesses and customize the role of the virtual attending to their community and resources.

## VIRTUAL REVIEW

Reviewing clinical cases and formulating management plans with PAs and medical trainees is a familiar role for many ED physicians. The level of involvement or intervention of the attending staff physician usually varies based upon the experience and comfort level of the trainee/PA, the case acuity or complexity, and the level of risk tolerance of the attending physician.

### Selecting a workforce

We used our PAs for supporting a virtual review model of care. Other providers that could be considered would be senior residents. Nurse practitioners have their own independent license, but may consult physicians if required and, thus, could also be used. Our PAs have a clear understanding of their scope of practice and their limitations. It is helpful (but not essential) that the on-site bedside provider and at-home virtual attending already have an established mutual trust.

Physician assistants are expected to work in a virtual review model of care provided on-site backup from an alternate staff physician working concurrently in the ED is available. Support from the on-site staff physician might include, for example, corroboration of a physical exam finding when indicated. The on-site staff physician also needs to be available to receive handover and become the Most Responsible Physician if patient complexity exceeds what can safely be managed virtually. Our “backup” physician is not explicitly stipulated; however, the PA typically finds a physical physician working the area of the ED where the patient was seen.

### Patient selection

Many patients are inappropriate for virtual review. These include patients with high levels of acuity or complexity, patients needing complicated procedures, and patients with problems that would fall outside the scope of practice of the physician assistant or trainee. At our center, the virtual attending and their delegate touch base at the beginning of a shift. The virtual attending shares responsibility for selecting appropriate patients for virtual review. Some lower acuity patients lend themselves well to a virtual review model, especially those presenting with mild illness or contact exposure and hoping only to be evaluated or tested for COVID-19 infection. In our department, most patients who undergo virtual review are Canadian Triage and Acuity Scale (CTAS) 4 or 5, have a relatively straightforward complaint, and do not require an in-depth physical exam. While we have approached patient selection cautiously given the novelty of this model, we are actively expanding to include other patients that require minimal physical examination (referred stable subspecialty patients, mental health patients with departmental social work support, etc).

### Communication with patients and/or families

An open discussion with families about the nature of virtual review is important. If families are uncomfortable with the arrangement, then that patient should be reviewed by the on-site staff physician, despite potentially contributing to longer wait times for other patients. We have documented verbal consent in the patient chart for virtual reviews. The patient or family may wish to speak with the virtual attending; staff may phone the patient/family's mobile phone to obtain further history or to provide nuanced patient education. After obtaining consent, we have also used video platforms, such as WhatsApp, FaceTime, and Zoom (the latter of which can be purchased in a PHIPA compliant version).

### Medicolegal risk

Each jurisdiction will need to clarify with their individual licensing body regarding any medicolegal risks associated with a virtual review workflow. During a serious health care crisis, such as a pandemic, most licensing and legal bodies would recognize the extenuating circumstances, which was the case in our jurisdiction. Medicolegal concerns are low provided that the clinician is acting in good faith, within their normal scope of practice, and with the support of their hospital leadership team (Canadian Medical Protective Association, personal communication, correspondence, March 23, 2020).^1,2^

PAs provide medical care for patients through physician delegation, which may include both written medical directives and direct orders.^3^ Each province has specific guidance available on the requirements and limitations of medical delegation. PAs are nonautonomous health care providers and must work under the supervision of a physician. Nearly all jurisdictions recognize that this supervision may be indirect with the supervising physician off-site.^4^ The PAs that work in our institution are also required to maintain certification with the Canadian Association of Physician Assistants and hold individual malpractice insurance to further mitigate medicolegal risks.

## TELEPHONE CALLS

Tertiary referral centres (and indeed many EDs) routinely receive phone calls from outside providers seeking transfer or medical advice. During the pandemic, our physicians have been less available to take phone calls when frequently donning and doffing PPE between patient rooms, along with the need to clean the phone after each use. The virtual attending has taken over these incoming calls, by having our unit clerk forward the calls to the virtual attending. At the end of the call, depending on the complexity of the case, the virtual physician may relay the information to the on-site physician or nursing team leader if required, otherwise, documentation is noted in our EMR system. This process is not used for critically unwell patients or trauma calls where arrival may be imminent and/or urgent management decisions are required. Although not done at our hospital, the virtual attending could expand their role depending on a given department's needs—they could take EMS patch calls, lead discussions from local long-term care homes, and consult more broadly with community providers.

## CALL-BACKS

Some EDs assign a specific clinician to be responsible for the communication of late-arriving significant results to patients who have already been discharged or diagnostic imaging discrepancies.^5,6^ In our ED, this workload is assigned to a specific on-shift clinician or designate. This workload has increased significantly, as our hospitals is now doing COVID-19 testing in-house. In an effort to provide patient-centered care, we have been notifying families of both positive and negative test results. This has created additional administrative burden to the providers facilitating these call-backs. Having the virtual physician at home who can manage these results by calling patients or families can significantly decrease the anxiety felt by patients and caregivers, and reduce the workload of the on-site team. They are also able to fix/change outpatient prescriptions, call back patients with positive blood cultures and update families with any diagnostic imaging discrepancies.

## CONCLUSION

As the COVID-19 pandemic evolves, it is clear that EDs can expect a certain level of staff attrition due to quarantines and illnesses, or due to voluntary exclusion of at-risk clinicians. By implementing creative staffing configurations, leveraging off-site access to EMRs and modern communication tools, these individuals can still contribute meaningfully to the operations of the department. By reviewing patients virtually, fielding phone calls, and helping with result call-backs, these clinicians can help decrease the burden on their frontline colleagues and also assist with conserving PPE.
